# Tumor and red bone marrow dosimetry: comparison of methods for prospective treatment planning in pretargeted radioimmunotherapy

**DOI:** 10.1186/s40658-014-0104-x

**Published:** 2015-02-24

**Authors:** Wietske Woliner-van der Weg, Rafke Schoffelen, Robert F Hobbs, Martin Gotthardt, David M Goldenberg, Robert M Sharkey, Cornelis H Slump, Winette TA van der Graaf, Wim JG Oyen, Otto C Boerman, George Sgouros, Eric P Visser

**Affiliations:** Department of Radiology and Nuclear Medicine, Radboud University Medical Center, P.O. Box 9101, 6500 HB Nijmegen, The Netherlands; Department of Radiology, Johns Hopkins University, Baltimore, MD USA; Immunomedics, Inc., Morris Plains, NJ USA; University of Twente, Enschede, The Netherlands; Department of Medical Oncology, Radboud University Medical Center, Nijmegen, The Netherlands

## Abstract

**Background:**

Red bone marrow (RBM) toxicity is dose-limiting in (pretargeted) radioimmunotherapy (RIT). Previous blood-based and two-dimensional (2D) image-based methods have failed to show a clear dose-response relationship. We developed a three-dimensional (3D) image-based RBM dosimetry approach using the Monte Carlo-based 3D radiobiological dosimetry (3D-RD) software and determined its additional value for predicting RBM toxicity.

**Methods:**

RBM doses were calculated for 13 colorectal cancer patients after pretargeted RIT with the two-step administration of an anti-CEA × anti-HSG bispecific monoclonal antibody and a ^177^Lu-labeled di-HSG-peptide. 3D-RD RBM dosimetry was based on the lumbar vertebrae, delineated on single photon emission computed tomography (SPECT) scans acquired directly, 3, 24, and 72 h after ^177^Lu administration. RBM doses were correlated to hematologic effects, according to NCI-CTC v3 and compared with conventional 2D cranium-based and blood-based dosimetry results. Tumor doses were calculated with 3D-RD, which has not been possible with 2D dosimetry. Tumor-to-RBM dose ratios were calculated and compared for ^177^Lu-based pretargeted RIT and simulated pretargeted RIT with ^90^Y.

**Results:**

3D-RD RBM doses of all seven patients who developed thrombocytopenia were higher (range 0.43 to 0.97 Gy) than that of the six patients without thrombocytopenia (range 0.12 to 0.39 Gy), except in one patient (0.47 Gy) without thrombocytopenia but with grade 2 leucopenia. Blood and 2D image-based RBM doses for patients with grade 1 to 2 thrombocytopenia were in the same range as in patients without thrombocytopenia (0.14 to 0.29 and 0.11 to 0.26 Gy, respectively). Blood-based RBM doses for two grade 3 to 4 patients were higher (0.66 and 0.51 Gy, respectively) than the others, and the cranium-based dose of only the grade 4 patient was higher (0.34 Gy). Tumor-to-RBM dose ratios would increase by 25% on average when treating with ^90^Y instead of ^177^Lu.

**Conclusions:**

3D dosimetry identifies patients at risk of developing any grade of RBM toxicity more accurately than blood- or 2D image-based methods. It has the added value to enable calculation of tumor-to-RBM dose ratios.

## Background

The aim of radioimmunotherapy (RIT) is to selectively target radioactivity to tumor lesions, with limited radiation dose to healthy tissues. The absorbed dose (AD) depends on the patient-specific pharmacokinetics of the tracer, the administered activity, and the radionuclide. After pre-therapeutic administration of a diagnostic-labeled compound, dosimetric calculations (matched pair dosimetry) lead to a patient-specific insight into how best to treat the patient. For example, pre-treatment dosimetry can be used to adjust the individual therapy dose, or even be used to select the most suitable radionuclide for therapy. Ideally, this will lead to an improved benefit-versus-risk ratio for individual patients.

In external beam radiotherapy, patient-specific treatment planning is a common practice because treatment planning is based on absorbed dose distributions and dose-response relationships for both tumor and normal tissues are relatively well known. However, for RIT, dose-response relationships have not been rigorously established and dose estimation has been less accurate and focused on calculating mean absorbed doses. In radionuclide therapy, the mean absorbed dose may not be useful for predicting tumor response and, in some cases, for predicting normal organ toxicity. Therefore, further investigation of dose-response relationships and development of dosimetry methods that provide dose-volume histograms and that incorporate radiobiological modeling for the clinical practice of radioimmunotherapy are highly desirable.

Since the red bone marrow (RBM) is often dose-limiting in RIT [1-6], the focus of the present study was on RBM dose calculation and the dose-toxicity relationship. Commonly used methods to calculate the RBM AD are the blood-based method, a two-dimensional (2D) image-based method, or a combination of these two methods. Although blood-based (or partially blood-based) dosimetry is an accepted method for estimation of the RBM dose [1,3,7-9], the correlation with the observed hematological toxicity is insufficient for clinical use [5,10]. Especially when the radiopharmaceutical shows RBM retention, 2D image-based dosimetry seems to be a better predictor for hematological toxicity than the blood-based method [5].

In our institution, both the abovementioned methods were prospectively applied in a clinical phase I pretargeted RIT study in 20 patients with advanced colorectal cancer [11]. Dosimetric data based on administration of a diagnostic ^111^In-labeled tracer were used to predict blood-based and 2D image-based RBM AD in a subsequent treatment with the same compound, but labeled with ^177^Lu. Although relatively low RBM absorbed doses (<0.31 Gy) were predicted in several patients, RBM toxicity was observed. According to the NCI Common Terminology Criteria v3, five patients developed grade 1 to 2 thrombocytopenia and two patients developed grade 3 to 4 thrombocytopenia, and three patients developed grade 1 to 2 leucopenia. Based on the applied dosimetry methods, no dose limit based on the ^177^Lu dosimetry could be defined that distinguishes the patients who showed toxicity from those without toxicity. Clearly, these calculations did not reliably predict toxicity in this experimental treatment. This indicates that an improved dosimetry method leading to doses correlating with the RBM toxicity and probably in the future use for prediction - and ideally prevention - of toxicity is desirable.

Furthermore, based on the data of the phase I study, it was suggested that treatment with ^90^Y might improve the tumor-to-RBM dose ratio [11]. The half-life of ^90^Y (2.66 versus 6.71 days for ^177^Lu) better corresponds with the peptide residence time in the tumor [11] and therefore should lead to a higher tumor-to-RBM ratio. In addition, ^90^Y is a well-known therapeutic radionuclide, e.g., as ^90^Y-ibritumomab tiuxetan (Zevalin®, Bayer Schering Pharma AG, Berlin, Germany), an FDA-approved drug for treatment of non-Hodgkin's lymphoma.

Assessment of the tumor-to-RBM dose ratio requires 3D dosimetry, since 2D image-based tumor dosimetry does not lead to reliable results as a result of overprojection with background tissue. Also, for the RBM dose calculation, 3D dosimetry is preferred since it has been shown that single photon emission computed tomography (SPECT)-based dosimetry leads to smaller errors than planar image-based dosimetry [12]. This is mainly due to the lack of overprojecting organs and more accurate attenuation correction.

Therefore, we hypothesized that 3D SPECT-based RBM dosimetry results better correlate with bone marrow toxicity than the blood-based and 2D image-based dosimetry results.

Recently, Boucek and Turner demonstrated a relationship between myelotoxicity and RBM dose, calculated using a 3D image-based dosimetry method in 23 patients with non-Hodgkin's lymphoma. They used a three-compartment model, comprising the blood, whole body, and RBM activity, which was measured in the spine, pelvis, and femur [13]. They calculated the RBM dose with a method relying on *S*-values, which are calculated using idealized representative model-based anatomical phantoms [14]. The use of idealized model-based *S*-values for individual patient anatomy may not account for important differences between the model-based representation and the individual patient anatomy. Such differences are particularly important in a therapeutic setting. Schwartz et al. [4], who also used an *S*-value-based method, did not find a correlation with toxicity. This emphasizes the potential importance of investigating dosimetry methods for RBM dosimetry that do not rely on idealized anatomical models and that are more likely to give results that correlate with RBM toxicity.

In this study, we applied a method for 3D RBM dosimetry that does not depend on *S*-values but instead uses Monte Carlo simulations for the dose calculation. For this purpose, the 3D radiobiological dosimetry (3D-RD) software [15,16] was used in patients who had undergone pretargeted RIT with a bispecific antibody and a ^177^Lu-labeled peptide. The aims were (1) to accurately estimate the RBM dose and the tumor dose, (2) to correlate the RBM dose with the RBM toxicity, and (3) to use the results for selection of the most suitable radionuclide for this therapy.

## Methods

### Patients

Patients with metastatic colorectal cancer for whom no standard treatment was available were eligible for the pretargeted RIT study. The protocol (ClinicalTrials.gov Identifier NCT00860860) was approved by the Regional Ethics Review Board (CMO). Written confirmed consent was obtained from all patients prior to any study-related procedures.

The patients received the anti-CEACAM5 × anti-hapten humanized trivalent bispecific antibody TF2. One day later, the ^177^Lu-labeled di-HSG peptide IMP288 (2.5 to 7.4 GBq) was administered as reported previously [11]. Of the 20 patients in this study, 13 were eligible for the 3D image-based dosimetry as they had undergone a series of four SPECT scans after administration of the ^177^Lu-labeled peptide.

### Image acquisition and use in dosimetry

Anterior and posterior whole-body planar images were acquired immediately (at 8 cm/min scan speed), at 3 h (6 cm/min), at 24 h, and at 72 h (both 4 cm/min) after administration of the ^177^Lu-labeled peptide, and followed by a SPECT scan involving continuous, circular scanning with a 180° scan arc, 64 views with both camera heads and 19 s/view. For each patient, a region for the SPECT scanning was selected that contained the kidney region and at least one tumor lesion, as well as at least two lumbar vertebrae (LV).

A Siemens dual-head gamma camera (ECAM, Siemens Medical Soluations, Hoffman Estates, IL, USA), equipped with medium-energy collimators was used with a symmetric 15% window over the 208-keV energy peak for SPECT scanning and an additional 15% window over the 113-keV peak for the planar images.

The planar ^177^Lu images were only used to calculate the ^177^Lu RBM dose. Due to overprojection of the background tissue, tumor dosimetry could not be performed on planar images.

Subsequently, the SPECT scans were used for tumor and RBM dosimetry of the ^177^Lu dose. Simulation of ^90^Y doses was performed (matched pair dosimetry) for comparison with the ^177^Lu tumor-to-RBM dose ratios. For the ^90^Y simulation, the SPECT images were rescaled to correct for the difference in half-life. Absorbed doses were calculated relative to the administered activity.

A contrast-enhanced diagnostic computed tomography (CT) scan and a fluorodeoxyglucose-positron emission tomography (FDG-PET)/CT (Biograph BGO duo, Siemens Medical Solutions, Knoxville, TN, USA) in accordance with the EANM guidelines were performed within 2 weeks prior to study entry, and for follow-up, 8 weeks after ^177^Lu injection [17].

### 2D image- and blood-based red bone marrow dosimetry

During the pretargeted RIT study, the dose to RBM was calculated using a blood-based [3] and a 2D image-based dosimetry method [18].

The blood-based RBM dose was based on blood samples collected 2 min, 30 min, 1 h, 2 h, 4 h, 24 h, and 72 h after peptide injection and on the total body activity (retrieved from the whole-body planar images). The blood samples were counted in a gamma counter (Wizard, Pharmacia-LKB, Uppsala, Sweden), using appropriate energy windows, with reference samples prepared from the injected products. Whole-body activities were calculated and combined with the blood counts for the final dose calculation as described by Shen et al. [3]. This was performed with the SPRIND software [18] using an RBM-to-blood activity concentration of 1, as was determined for ^177^Lu-peptide [19].

For the 2D image-based method, SPRIND was used to delineate the cranium, representing the activity in the RBM and to calculate the time-integrated activity coefficients. The time-integrated activity coefficient of the cranium activity was divided by the fraction of the RBM mass in the cranium to the RBM mass in the total skeleton, for which the default value of 0.119 was taken from ICRP23's reference man. Subsequently, OLINDA software [20] was used to calculate the RBM dose.

### SPECT image reconstruction

SPECT scans were reconstructed using the iterative reconstruction software ReSPECT (Scivis, Göttingen, Germany), in six iterations, without noise reduction, with background subtraction and attenuation correction using an attenuation coefficient of 0.13 cm^−1^, based on the results of Brown et al. [21]. The algorithm uses attenuation correction with constant attenuation coefficient within body contours estimated from photopeak and scatter projections.

This implies that a correct body contour is needed for quantitative evaluation of the images. In ReSPECT, the ‘threshold object background’ is used for definition of the body contour; voxels with a value higher than this threshold are assigned to the body. The contour can be defined correctly by the default threshold as long as the target (body)-to-background activity ratio is high enough, which was the case for the first two SPECT scans. For the third and fourth scans, the contour was fitted to the contour of the first scan by lowering the threshold value, as the lower whole-body activity would have led to a smaller body contour at the default settings. The comparison of body contours of the early and later SPECT scans was performed with MATLAB (MathWorks, Natick, MA, USA).

### Calibration

To convert the pixel values in the SPECT images to activity (Bq), a calibration experiment was performed. A cylindrical phantom was filled with 440 MBq ^177^Lu dissolved in 9.1 L water and imaged using the same scanning and reconstruction protocol as used for the patient SPECTs. The known activity at the time of acquisition, combined with the number of counts in a volume drawn around the phantom, resulted in a calibration factor of 6.23 × 10^−6^ MBq per count. In a separate calculation, we estimated the dead time typically present in patient scans and in the phantom scan, using the equations that hold for paralyzable system as described in Cherry et al. [22]. The observed values based on quality control measurements of the SPECT camera, using the ‘two-source method’, were in the order of 0.5 to 2 μs. This led to errors in activity concentrations at a maximum of 1%. For this reason, we did not correct the images for the dead time.

### Delineation and co-registration

Tumor-oriented co-registration of the SPECT and the low-dose CT images was performed using the HERMES Gold 2.10 software (HERMES Medical Solutions, Stockholm, Sweden). The co-registered CT images were cropped and scaled to the same matrix size (128 × 128 × 78) and voxel dimensions (4,795 × 4.795 × 4.795 mm) as the SPECT images. For each patient, VOIs were manually delineated in the LV (VOI_LV_) on the SPECT images for RBM dosimetry and for one tumor lesion (VOI_tumor_) on the co-registered CT images for the tumor dosimetry.

For the VOI_LV_, at each time point, the RBM containing parts of at least two LV were delineated and further processed as one VOI. If delineation on the SPECT image was not possible because the LV could not be distinguished from the background, the co-registered CT image was used for delineation.

Tumors were delineated on the rescaled CT by using a SPECT overlay. Occasionally, after checking the VOIs on the PET images, the regions were changed so that FDG-positive areas of tumors that were SPECT-negative were included in the delineation because FDG-PET and SPECT images show different aspects of the pathophysiology. Necrotic cores were included in the VOI, since these areas might still contain vital tumor cells.

### 3D-RD

For the 3D dosimetry, the Monte Carlo-based 3D-RD dosimetry package - developed at the Johns Hopkins Medical Institute (Baltimore, USA) - was used [16,23]. In this software, the co-registered low-dose CT scan is used to assign a density value and composition (soft tissue, lung, or bone) to each voxel in the corresponding SPECT scans. The density, composition, and the activity maps were the input for the Monte Carlo (MC) simulations. For each SPECT scan, 10^6^ MC simulations were run, using the spectra probability distributions obtained from MIRD [24], resulting in a dose rate value per voxel for each scan.

The dose per voxel was calculated by integrating the dose rates for each voxel, using a hybrid trapezoidal-exponential fit consisting of linear fits between the first three time points and an exponential tail obtained by fitting to the final two time points [25]. This finally resulted in a dose map. The VOI mean absorbed doses were obtained by summing the deposited energy in the VOIs and integrating these resulting VOI dose rates. For this integration, a similar fit as for the voxel doses was used.

#### 3D tumor dosimetry

For the VOI_tumor_, the mean absorbed dose (AD_tumor_) and a dose-volume histogram (DVH) were calculated using 3D-RD. The DVH provides insight into the spatial distribution of AD in the tumor. For comparison with the AD_tumor_, the AD in the VOI_tumor_ was also calculated using the sphere model as incorporated in OLINDA [20]. For this purpose, the mass of the VOI was calculated using the density map created from the low-dose CT and was used as the sphere mass as required by OLINDA. The tumor time-integrated activity coefficient was calculated using the total activity in the VOI_tumor_ at the four different time points, again using a similar hybrid trapezoidal-exponential fit.

#### 3D LV-based RBM dosimetry

The rigid co-registration as described above was tumor-oriented, and it was observed that this did not lead to adequate co-registration of the LV, especially when slight differences in bending of the spine were observed. Therefore, delineation of the LV VOI was performed on each SPECT image separately, precluding the ability to perform voxelized dosimetry, and thus, only mean dose rates and mean absorbed doses were calculated for the VOI_LV_ (AD_RBM_).

In addition to AD_RBM_, the biological effective dose (BED_RBM_) was also calculated:

1 $$ {\mathrm{BED}}_{\mathrm{RBM}} = {\mathrm{AD}}_{\mathrm{RBM}}\left(1+\frac{G\left(\infty \right)}{\alpha /\beta }{\mathrm{AD}}_{\mathrm{RBM}}\right) $$

with *α* and *β* as the radiobiological parameters from the linear quadratic equation model (*α*/*β* = 10 Gy [26]), AD as the absorbed dose, and *G*(∞) as the Lea-Catcheside G-factor expressing the reduction in cell kill as a result of sublethal damage repair during treatment and which depends on the DNA repair rate (*μ*) = 0.46 h^−1^ [26].

The 3D LV-based AD_RBM_ was compared to the blood-based and the 2D cranium-based RBM dosimetry results and correlated with the grade of thrombocytopenia (graded according to NCI Common Terminology Criteria v3), since thrombocytopenia was the major toxicity experienced in this study.

### Tumor-to-RBM dose ratios

In adequate treatment settings, the AD_tumor_ should be sufficiently higher than the AD_RBM_. To evaluate this, the tumor-to-RBM dose ratio AD_tumor_/AD_RBM_ was calculated. This calculation was done for the ^177^Lu dose and the simulated ^90^Y dose.

## Results

### Patients, VOIs, and RBM toxicity

**Figure 1:**
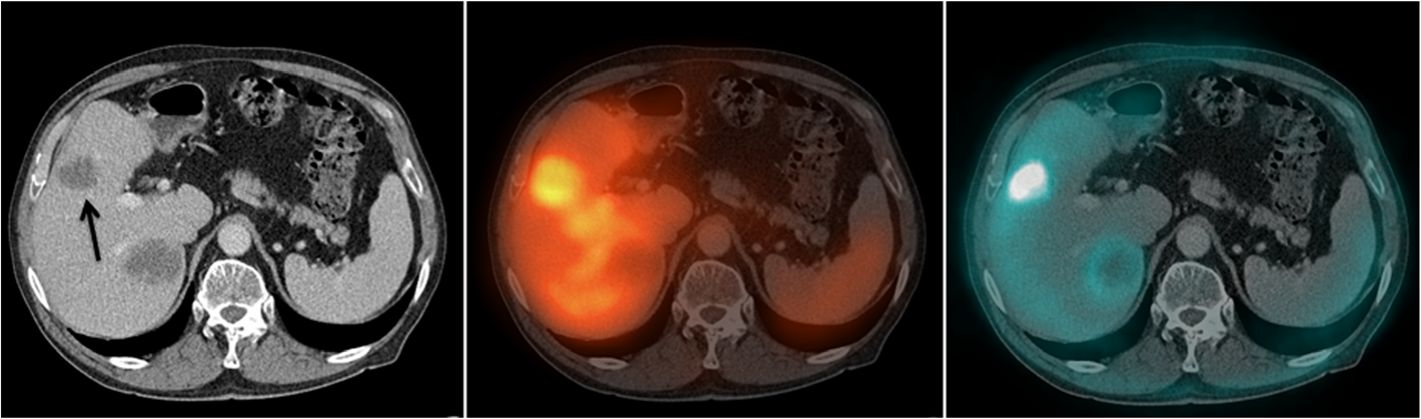
**Metastases in the liver of patient 10 showing the lesions on CT (left),** ^**177**^ **Lu-peptide SPECT/CT (middle) and FDG-PET/CT (right).** The arrow indicates the lesion that was used for the tumor dosimetry.

Table 1 summarizes patient and VOI characteristics, the calculated mean tumor and RBM doses, and the grades of thrombocytopenia and leucopenia, representing the severity of the RBM toxicity. Patient numbers are the same as those used in the report of the phase 1 study [11]. Figure 1 shows an example of a tumor lesion in the liver on CT, SPECT, and PET images.

**Table 1:** **Pa** **tient and VOI**_tumor_ **details and dose results**

**Pt. #**	**Age (years)**	**Gender**	**Location of VOI**_**tumor**_	**AA (GBq)**	**Volume of VOI**_**tumor**_ **(mL)**	**tox**	**3D-RD**		**OLINDA**
						**grade thr - grade leuc**	**AD**_**tumor**_ **(Gy)**	**AD**_**RBM**_ **(Gy)**	**AD**_**tumor**_ **(Gy)**
7	63	F	Rectum	6.2	14	0 - 0	0.73	0.39	0.71
10	70	M	Liver	7.4	70	4 - 1	2.94	0.51	2.67
11	55	F	Liver	7.4	44	0 - 0	2.53	0.29	2.47
12	70	M	Liver	7.4	386	0 - 0	1.54	0.30	1.47
13	76	M	Colon	7.4	23	0 - 0	0.51	0.12	0.51
14	52	F	Liver	4.0	23	1 - 0	3.70	0.70	3.72
15	58	M	Liver	5.9	285	1 - 0	0.86	0.58	0.83
16	76	M	Colon	4.6	121	3 - 2	0.66	0.97	0.64
17	73	M	Lung	4.5	65	2 - 0	0.63	0.43	0.62
18	63	F	Liver	2.5	104	0 - 2	4.52	0.47	4.44
19	66	F	Liver	7.4	141	0 - 0	1.45	0.28	1.38
20	72	M	Lung	5.6	723	1 - 0	0.46	0.72	0.45
21	39	F	Liver	5.6	154	0 - 0	2.47	0.23	2.45
Mean	64				166		1.77	0.46	1.72

RBM = red bone marrow. AA = administered activity, Pt. # = patient number, tox = toxicity, grade thr = grade thrombocytopenia, grade leuc = grade leucopenia.

### 3D tumor doses

**Figure 2:**
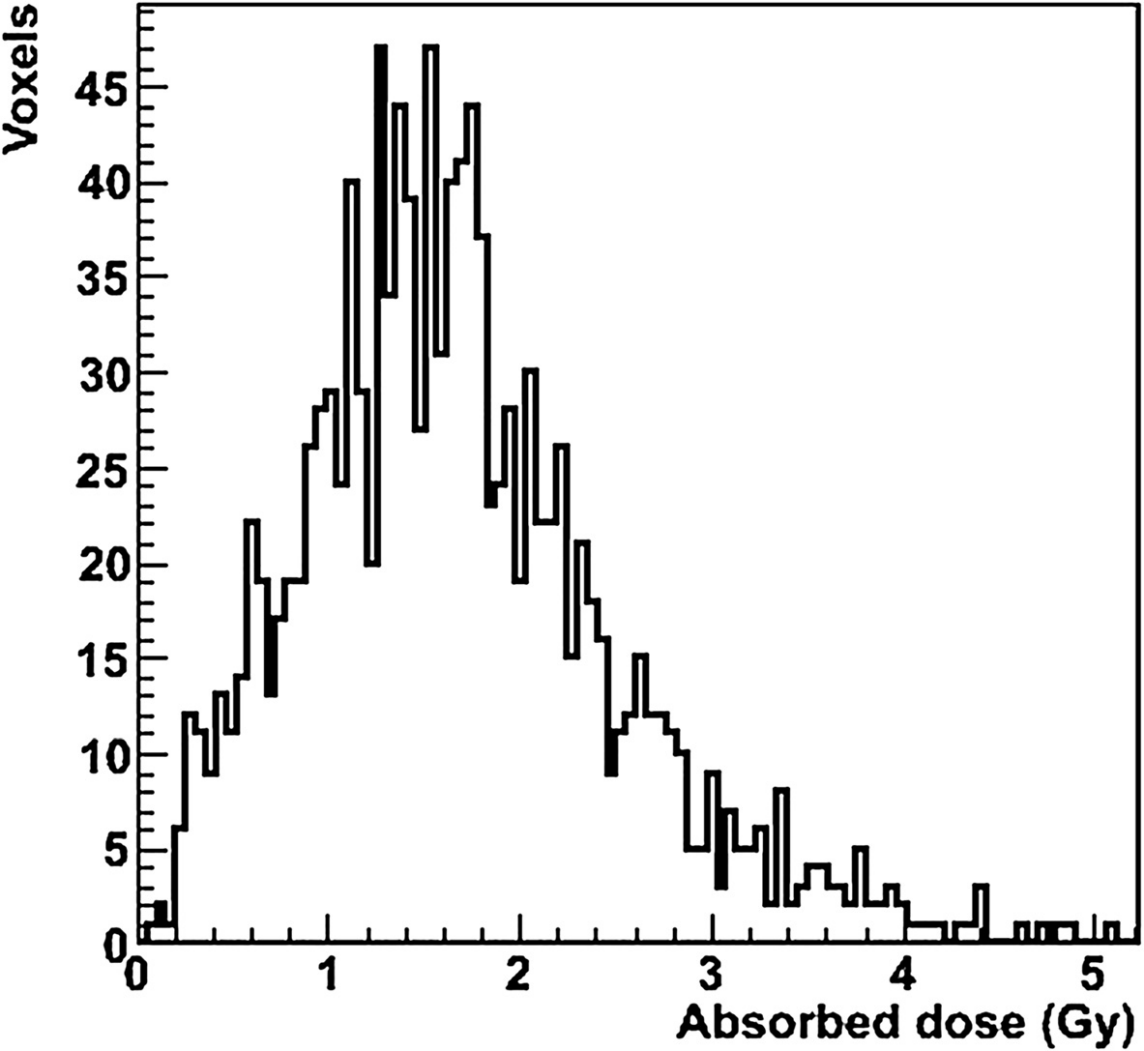
**Dose-volume histogram of the tumor volume of interest, located in the liver of patient 19.**

As shown in Table 1, the ^177^Lu AD_tumor_ are 0.46 to 4.52 Gy. A typical DVH of a tumor is shown in Figure 2. The AD_tumor_ calculated with 3D-RD was systematically greater than the OLINDA AD, although in most patients, the difference was small (difference <5.0%), except for one patient (number 10) who showed a 9.6% higher 3D-RD tumor dose.

In all patients, the BED was just slightly higher than the AD with a maximum difference of 0.11%, which is expected due to the relatively low AD values.

### Correlation between RBM dose and RBM toxicity

**Figure 3:**
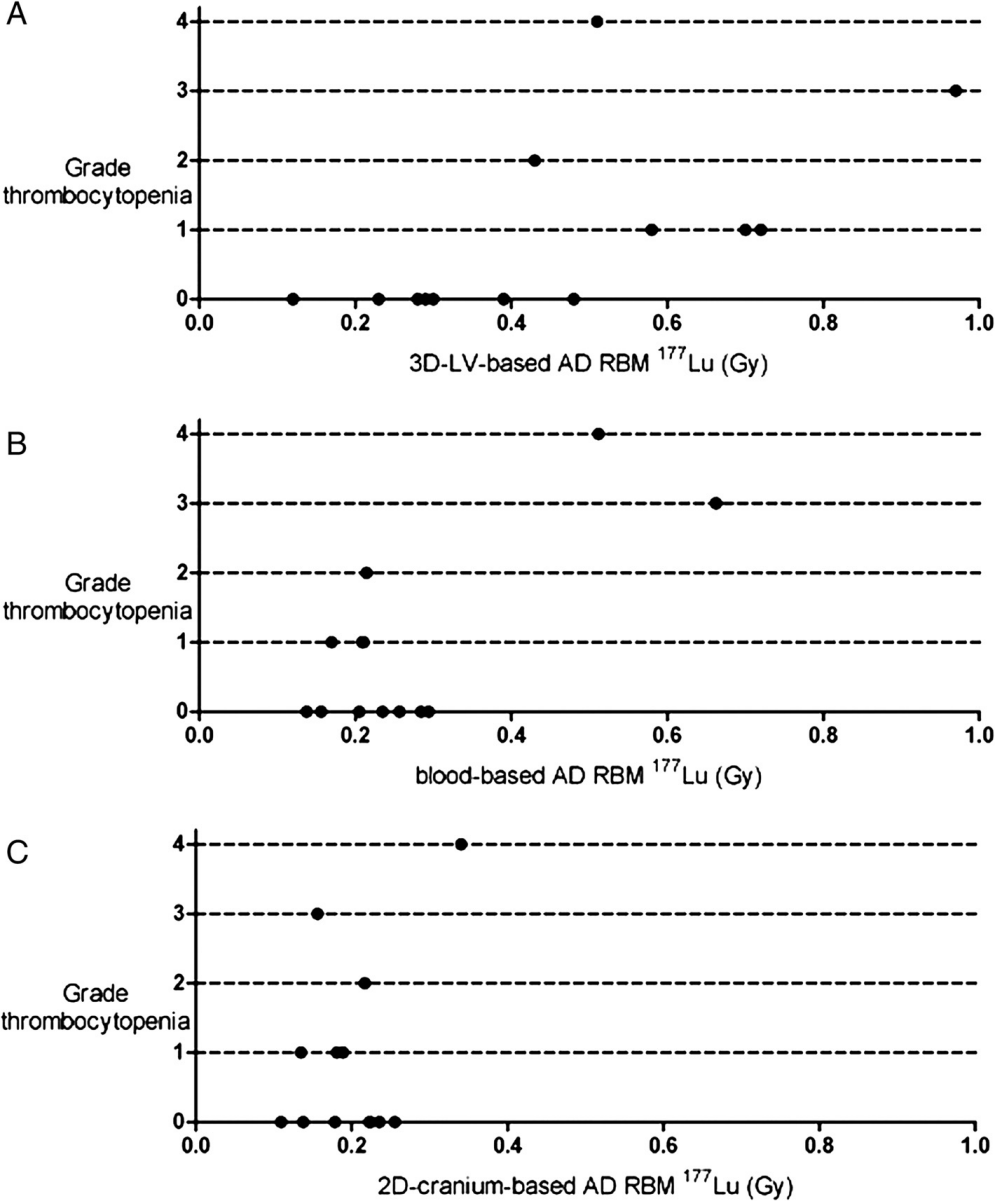
**3D-RD-based (A), blood-based (B), and 2D cranium-based (C) absorbed dose in the red bone marrow versus the grade of platelet toxicity.** AD RBM = absorbed dose red bone marrow.

Figure 3 shows the blood-based, cranium-based, and 3D-RD-based RBM doses versus the grade of thrombocytopenia. 3D-RD-based RBM doses (Figure 3A) were generally higher than the doses calculated with the blood-based and 2D image-based methods (Figure 3B,C, respectively). The highest 3D-RD-based RBM dose without thrombocytopenia and/or leucopenia was 0.39 Gy. The other patients showed RBM toxicity and had RBM doses in the range of 0.42 to 0.97 Gy. There was only one patient in this range (0.47 Gy) without thrombocytopenia, but this patient had a grade 2 leucopenia.

As shown in Figure 3B,C, the blood-based and cranium-based methods do not separate patients with and without any grade of toxicity. Based on the blood-based RBM dose, only patients with grade 3 to 4 thrombocytopenia could be distinguished (0.51 and 0.66 Gy) from the other patients (0.14 to 0.29 Gy). For the cranium-based dosimetry, the patient with grade 4 thrombocytopenia was the only patient with toxicity who showed a dose outside the AD range of the patients without toxicity (0.34 versus 0.11 to 0.26 Gy for the others).

**Figure 4:**
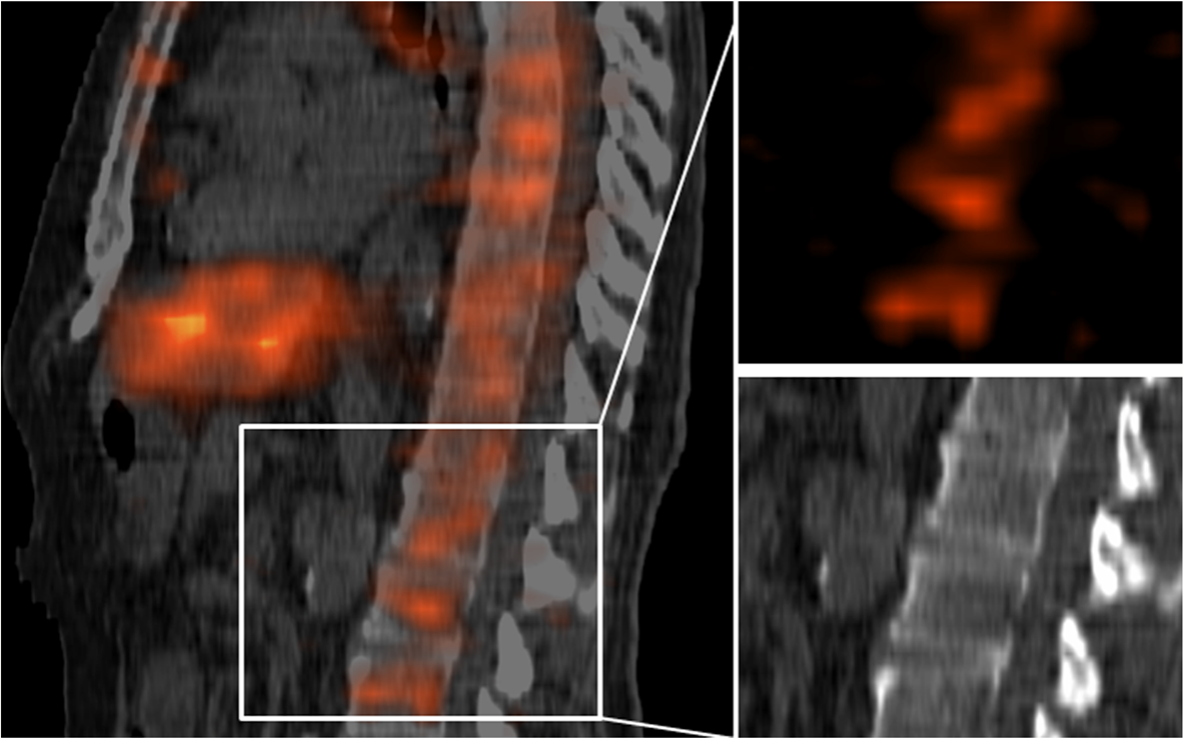
**Accumulation of activity in the RBM.** (Left) The sagittal cross section of low-dose CT and the SPECT image made at 72 h after administration of the ^177^Lu-peptide of patient 20. (Right) The SPECT and low-dose CT images of vertebrae in which activity accumulated.

Some patients showed accumulation of activity in the RBM (see Figure 4), leading to a higher RBM dose.

### Tumor-to-RBM dose ratios for ^177^Lu compared to ^90^Y

The effect of ^90^Y versus ^177^Lu was shown by the simulated ^90^Y dosimetry (see Table 2). The mean increase in the tumor-to-RBM dose ratio is 25% when using ^90^Y instead of ^177^Lu, and the mean ratio for the group changed from 4.68 for ^177^Lu to 5.41 for ^90^Y.

**Table 2:** **The difference between AD**_**tumor**_ **/AD**_**RBM**_ **for treatment with** ^**177**^ **Lu and** ^**90**^ **Y**

**Patient number**	**Ratio tumor/RBM dose**		**Percentage difference**
	^**177**^ **Lu**	^**90**^ **Y**	
7	1.88	2.13	13%
10	5.72	6.54	14%
11	8.84	9.78	11%
12	5.08	7.27	43%
13	4.33	4.33	0%
14	5.26	6.37	21%
15	1.49	2.29	54%
16	0.68	1.06	56%
17	1.46	2.02	39%
18	9.45	8.87	−6%
19	5.11	4.97	−3%
20	0.64	0.99	54%
21	10.91	13.72	26%

A positive difference represents a higher tumor-to-RBM dose ratio in ^90^Y, and the percentage is calculated compared to the ^177^Lu ratio. RBM = red bone marrow.

**Figure 5:**
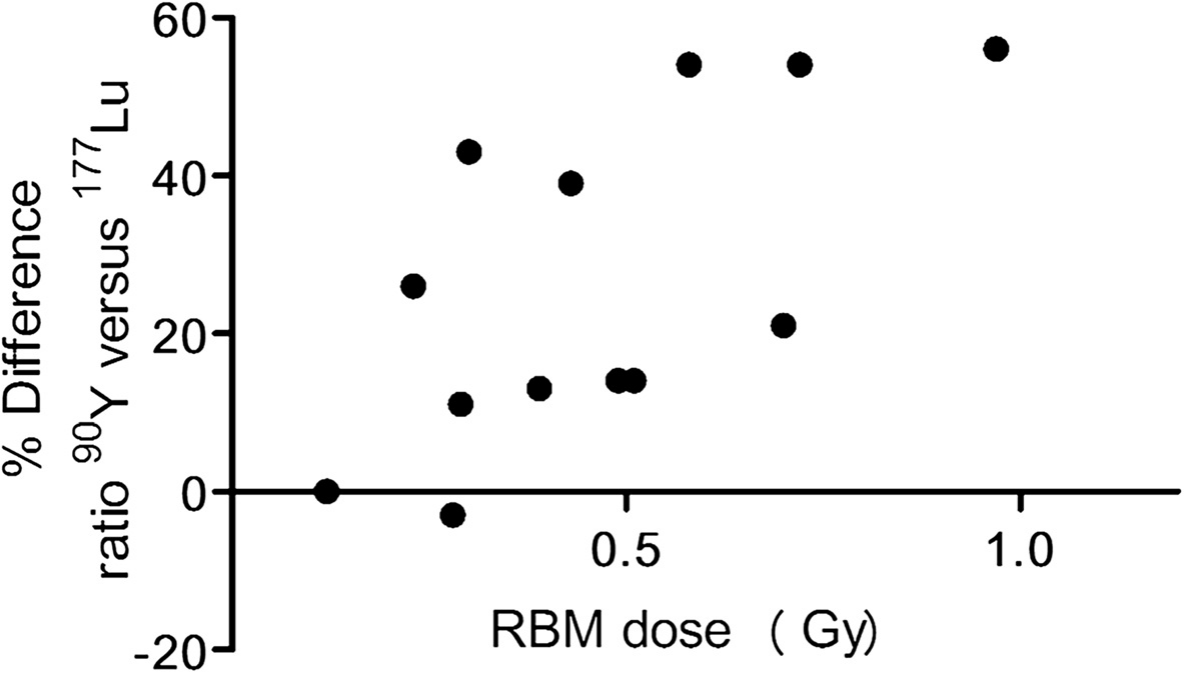
**Red bone marrow (RBM) dose versus the percentage difference in AD**_tumor_ **/AD**_RBM_ **for** ^**90**^ **Y compared to** ^**177**^ **Lu.** A positive difference represents a higher tumor-to-RBM dose ratio in ^90^Y.

Interestingly, patients with a high RBM dose in ^177^Lu treatment showed the largest percentage increase in the tumor-to-RBM dose ratio when changing to ^90^Y (Figure 5)

.

## Discussion

In this study, we demonstrated the utility of 3D-RD for tumor and RBM dose calculation and the correlation with RBM toxicity.

Using 3D-RD, all patients with RBM toxicity had a higher RBM dose than the patients without any RBM toxicity. In contrast, this was not observed with the conventional blood-based and 2D cranium-based methods which might be caused by an underestimation of the calculated doses.

RBM doses were in most patients higher when calculated with 3D-RD than those with the blood-based method (11/13 patients) and the cranium-based method (12/13 patients). In the blood-based RBM dosimetry used, it was assumed that the blood activity concentration is proportional to the RBM concentration at all time points. In most patients, we clearly observed RBM retention at the later imaging time points (24 and 72 h post injection). Therefore, at least in these patients, the blood-based method will result in an underestimation for the AD_RBM_ and therefore in a poorer correlation with the RBM toxicity, compared to the 3D-RD result.

The 2D cranium-based dosimetry might also have resulted in an underestimation of the AD_RBM_. The fraction of RBM in the cranium is used to represent the activity in the total RBM. In the SPRIND software package, this fraction was defined as 0.119, based on the ICRP23's reference man, representing a 40-year-old male. As the median age of our patient population was 63 years, this fraction might have been an overestimation. Since the distribution of the RBM over the skeleton is both age- and patient-specific, ideally, this fraction should be individually measured, and a patient-specific percentage of the RBM in a certain VOI should be used for this method.

For both methods, a patient-specific difference in the severity of the underestimation might explain the relative difference between the methods in relation to the observed RBM toxicity.

In addition, the difference between the 3D LV-based and 2D cranium-based AD_RBM_ could be explained by inhomogeneous uptake in the RBM [2] and the influence of activity surrounding the LV on the AD_LV_. Taking this into account, the 3D image-based results seem to be more accurate by avoiding the influence of overlapping organs and due to more accurate VOI drawing because the target-to-background ratio is higher for 3D than that for 2D.

Figure 3A suggests that RBM toxicity can be expected for RBM doses higher than 0.4 Gy. As in all dosimetry studies, the absolute value (i.e., 0.4 Gy) is highly influenced by methodological choices such as not to correct for the partial volume effect. This might partially explain the difference with the general rule that a blood (and RBM dose) of 2 Gy is considered to be safe [6,27,28]. Also, and probably most important, all patients had a history of multiple lines of polychemotherapy, which most certainly negatively affected bone marrow reserves and thus myelotoxicity [29].

For actual definition of a threshold, a larger study, including patient groups with and without previous chemotherapy, should be performed.

In this study, attenuation correction was performed using a method that assumes a homogeneous body density and therefore overestimates the attenuation in low-density tissue like the lungs. To avoid this, CT-based attenuation correction would be preferred for future studies. Unfortunately, there was no SPECT-CT available at our department during this clinical study that was finished in 2011.

In our view, the absorbed dose - or a derived parameter of the AD (i.e., BED) - has the potential to be predictive for different radiolabeled therapeutics, irrespective of the radionuclides, radiolabeled peptides, radiolabeled antibodies, or pretargeting. As we have shown, 3D image-based dosimetry seems to result in a clear dose-toxicity relationship and therefore might be a valuable approach for the calculation of a maximally tolerated absorbed dose (MTAD). However, a robust definition of a reliable MTAD based on this method would require processing of larger datasets.

The OLINDA-based results of the tumor-absorbed doses were comparable to the 3D-RD results and are in accordance with previous research [30]. In general, the OLINDA sphere model led to a somewhat lower dose. This could be explained by the influence of activity surrounding the VOI. For the use of the OLINDA sphere model, the VOI is assumed to be isolated. In 3D-RD, the surrounding activity does contribute to the tumor dose, which is more realistic. Nevertheless, we conclude that OLINDA provides a fast and practical method for tumor dosimetry.

OLINDA RBM dosimetry, or more generally S-value-based RBM dosimetry, is not feasible without a known (or a model-based) RBM mass. Using a dose point kernel as alternative for the MC-simulation would reduce the time needed for the calculation. Nevertheless, 3D-RD can easily provide dose rates per time point and enables delineation for each time point. Therefore, for the calculation of the bone marrow dose, we prefer 3D-RD and we consider MC-based software advantageous for RBM dosimetry, despite the fact that it is more time-consuming.

Matched pair dosimetry, as shown for ^177^Lu and ^90^Y, proved to be a convenient and inexpensive method (compared to animal or patient studies) for exploring the effect of the different radionuclide on the tumor-to-RBM dose ratio. The results of the ^90^Y simulation confirm the hypothesis that replacing ^177^Lu by ^90^Y would lead to an increase in the tumor-to-RBM dose ratio, especially in patients given a higher RBM dose. In such patients, we see a relatively slow, but clear, accumulation of activity in the RBM, which is most visible on the later scans. Therefore, the shorter half-life of ^90^Y is advantageous.

## Conclusions

3D-RD-based RBM dosimetry is feasible and a more sensitive predictor for selection of patients showing any grade of RBM toxicity than blood-based and planar image-based RBM doses. In addition, comparing the ^177^Lu tumor-to-RBM dose ratios with the simulated ^90^Y ratios proved to be a useful method to explore the effect of using a different radionuclide for this therapeutic modality.
